# Sine scleroderma, limited cutaneous, and diffused cutaneous systemic sclerosis survival and predictors of mortality

**DOI:** 10.1186/s13075-021-02672-y

**Published:** 2021-12-07

**Authors:** Sébastien De Almeida Chaves, Tiphaine Porel, Mickael Mounié, Laurent Alric, Léonardo Astudillo, Antoine Huart, Olivier Lairez, Martin Michaud, Grégoire Prévot, David Ribes, Laurent Sailler, Francis Gaches, Daniel Adoue, Gregory Pugnet

**Affiliations:** 1 Department of Internal Medicine, CHU Toulouse, Toulouse, Midi-Pyrénées France; 2 INSERM UMR1027, 37 Allées Jules Guesdes, Toulouse, Midi-Pyrénées France; 3 Universite Toulouse III Paul Sabatier Toulouse, Occitanie, France; 4 Department of Internal Medicine, Saint Exupéry Nephrology Clinic, Toulouse, Midi-Pyrénées France; 5 Department of Nephrology, CHU Toulouse, Toulouse, Midi-Pyrénées France; 6 Department of Cardiology, CHU Toulouse, Toulouse, Midi-Pyrénées France; 7 Department of Internal Medicine, Hospital Joseph Ducuing, Toulouse, France; 8 Department of Pneumology, CHU Toulouse, Toulouse, Midi-Pyrénées France

**Keywords:** Autoimmune diseases, Systemic sclerosis, Mortality, Prognostic factors

## Abstract

**Background:**

Systemic sclerosis (SSc) is associated with a variability of mortality rates in the literature.

**Objective:**

To determine the mortality and its predictors in a long-term follow-up of a bi-centric cohort of SSc patients.

**Methods:**

A retrospective observational study by systematically analyzing the medical records of patients diagnosed with SSc in Toulouse University Hospital and Ducuing Hospital. Standardized Mortality Ratio (SMR), mortality at 1, 3, 5, 10, and 15 years of disease and causes of death were described. Predictors of mortality using Cox regression were assessed.

**Results:**

Three hundred seventy-five patients were included: 63 with diffuse cutaneous SSc, 279 with limited cutaneous SSc, and 33 with sine scleroderma. The SMR ratio was 1.88 (95% CI 1.46–1.97). The overall survival rates were 97.6% at 1 year, 93.4% at 3 years, 87.1% at 5 years, 77.9% at 10 years, and 61.3% at 15 years. Sixty-nine deaths were recorded. 46.4% were SSc related deaths secondary to interstitial lung disease (ILD) (34.4%), pulmonary hypertension (31.2%), and digestive tract involvement (18.8%). 53.6% were non-related to SSc: cardiovascular disorders (37.8%) and various infections (35.1%) largely distanced those from cancer (13.5%). Four significant independent predictive factors were identified: carbon monoxide diffusing capacity (DLCO) < 70% (HR=3.01; *p*=0.0053), C-reactive protein (CRP) >5 mg/l (HR=2.13; *p*=0.0174), cardiac involvement (HR=2.86; *p*=0.0012), and the fact of being male (HR=3.25; *p*=0.0004).

**Conclusion:**

Long-term data confirmed high mortality of SSc. Male sex, DLCO <70%, cardiac involvement, and CRP> 5mg/l were identified as independent predictors of mortality.

**Supplementary Information:**

The online version contains supplementary material available at 10.1186/s13075-021-02672-y.

## Abstract (article highlights)

• Male sex, cardiac involvement, DLCO <70%, and CRP > 5 mg/l are strong predictors of mortality in systemic sclerosis.

• This study shows the survival of subtypes and in particular sine scleroderma.

• Sine scleroderma subtype has better survival than diffuse or limited cutaneous subtypes.

• Non-systemic sclerosis-related deaths are more frequent than systemic sclerosis-related deaths.

• Cardiovascular events non-systemic sclerosis-related are the main deaths.

**Supplementary Information:**

The online version contains supplementary material available at 10.1186/s13075-021-02672-y.

## Key messages

- One of the first studies that ascertained sine scleroderma mortality rate versus diffuse and limited subtypes
- 4 predictors of at 5, 10 years, and all mortality: male sex, cardiac involvement, DLCO <70%, and CRP > 5 mg/l.
- Importance of cardiovascular causes in non-systemic sclerosis-related death

## Introduction

Systemic sclerosis (SSc) is a severe systemic autoimmune connective tissue disease characterized by an elevated standardized mortality ratio (SMR) of 1.34 to 7.18 [1–17]. Because the presentation and prognosis of SSc are highly heterogeneous, studies showed a 10-year survival rate between 50 and 84% [4, 18–21].

Such mortality is still related to SSc in 27 to 72% of cases [3, 6, 8, 16–18, 21–27]. The two main causes of death remain interstitial lung disease (ILD) and pulmonary hypertension (PH). However, the SSc-non-related causes of death are increasing, particularly cardiovascular diseases, and infections [3, 6, 8, 15, 18, 19].

Assessment of prognosis is crucial to identify the patient who may benefit from close monitoring and immunosuppressants or autologous hematopoietic stem cells transplantation [20]. Given the wide variability of mortality rate reported in the literature, it appeared essential to obtain a better understanding of SSc prognosis and its associated risk factors in a well-characterized incident SSc cohort. The objective of the present study was to estimate mortality in the Systemic Scleroderma Toulouse Cohort (SSTC) in order to determine risk factors and causes of death.

## Methods and materials

### Data source

Data were obtained from the SSTC which includes incident patients with SSc who fulfilled the 2013 ACR/EULAR criteria and 2001 LeRoy and Medsger classification [28, 29], with retrospective collection of data between January 1, 1978, and May 30, 2018, and prospective onwards. The SSTC is a bi-centric cohort from the Toulouse University Hospital, a tertiary referral center for SSc, and Joseph Ducuing Hospital, a secondary referral center. Patients were selected through the coding system PMSI (Programme de Médicalisation des Systèmes d’Informations) and through the Doctors’ active patient files. Concerning the coding system PMSI, International Classification of Diseases, Tenth Revision (ICD-10) was used (M34, M34.1, M34.8, M34.8+J99.1, M34.8+ G73.7, M34.9), as we described in a previous study [30].

A thorough medical chart review followed by the entry of the standardized data collection form, for all consecutive unselected incident SSc patients were performed. SSc sub-types were classified as “diffuse SSc,” “limited SSc,” and “SSc sine scleroderma” [31]. Localized scleroderma patients (morphea and linear disease) were not included in the SSTC. The standardized data collection covered demographic aspects, disease duration, organ involvement, laboratory data, and drug exposure. Patients with more 25% of missing data were excluded. Disease onset was defined as the date of the first non–Raynaud’s phenomenon symptom attributable to SSc. Annual follow-up examinations were carried out. The forms were filled out by SSc specialists. Patients gave informed consent to participate in the SSTC. The data at baseline and at each follow-up evaluations (each annual follow-up and/or acute complication) were collected in the SSTC as part of routine clinical care in accordance to Good Clinical Practice and complied with the requirements of the Commission Nationale Informatique et Liberté (CNIL) (registration No. 914607). In compliance with French regulation relating to clinical non-interventional research, this study does not require ethics committee approval.

### Population

For the present study, we included adult (≥18 years) incident SSc subjects who had at least one follow-up visit during the first years in the SSTC and a disease onset between January 1, 2000, and January 1, 2018. Patients were followed until May 31, 2018.

### Collected data

Data collected at the inclusion visit were sex, ethnic group, age at disease onset, date of the first Raynaud’s phenomenon symptom, date of the first non–Raynaud’s phenomenon symptom, body mass index (BMI), smoking habits, SSc sub-types according to Leroy and Medsger [31], presence of arthralgia, myalgia, calcinosis or tendon friction rubs [32], gastrointestinal (GI) complications [33], neurological involvement [34], skin involvement as measured by the Rodnan modified skin score (mRSS) [35], and cardiac [36] and pulmonary evaluation [37], including pulmonary function tests (Forced Vital Capacity (FVC), diffusing capacity of the lung for carbon monoxide (DLCO)) according to the American Thoracic Society and European Respiratory Society (ATS/ERS) consensus standards [38], presence of SSc-related interstitial lung disease (ILD) or pleural effusion on chest X-ray or on high resolution computed tomography (HRCT), results from transthoracic echocardiography (TTE) including left ventricular ejection fraction (LVEF), tricuspid regurgitation velocity (TRV, m/s), and pulmonary arterial systolic pressure (PASP, mmHg) measurement [39]. Pulmonary arterial hypertension (PAH) was confirmed as a mean PAP ≥25 mmHg and was considered a SSc-associated pre-capillary PAH when associated with a pulmonary artery wedge pressure (PAWP) ≤ 15 mmHg [40]. Cardiac involvement was defined by LVEF <50%, and/or TTE abnormalities (pericarditis, cardiac valvulopathy, or diastolic dysfunction), and/or an electrocardiogram (ECG) abnormality (arrhythmia or conduction blocks). Atrial and ventricular arrhythmia were included in arrythmia. Any cardiac symptoms appearing prior to SSc diagnosis were considered as having no connection with the disease under study. The scleroderma renal crisis (SRC) was defined as a new onset of significant systemic hypertension (>150/85 mmHg) and acute renal failure (≥30% reduction in estimated glomerular filtration rate) [2, 41–44]. Laboratory parameters collected were hemoglobin level, serum creatinine, C-reactive protein, albumin, antinuclear (ANA), anti-centromere (ACA), anti-SCL70, anti RNA polymerase III, anti PM/Scl, anti-TIF1-y, and ANCA [45, 46]. If the clinical information was missing, this information was counted as missing data.

### Mortality data

Survival status was ascertained up until the end of this study based on the records in the database and systematically verified in each medical chart, telephone tracing of patient’s general practitioner (GP), telephone tracing of patients in whom no data had been entered for ≥24 months in the database, and systematic interrogation of either the Registre d’état civil [47]. The final status of loss to follow-up was defined as one where no data had been entered for ≥ 24 months with a failure to contact the patient or his GP despite at least two attempts, no death recorded in either the Registre d’état civil [47] and in French database recording deaths “https://deces.matchid.io/search.”

### Calculation of standardized mortality ratio

The SMR and its 95% confidence interval (95% CI) were calculated according to the ratio of observed death in the cohort to the number of deaths of the French age/sex-matched population [48]. The mortality rates of the general population were obtained from the French National Statistical Agency (Institut national de la statistique et des études économiques - INSEE), and the most recent available data at the time of data analysis were from December 2016 [49]. In relation to subjects lost to follow-up, we performed 2 sensitivity analyses to recalculate SMR, one of which assumed that all such subjects were alive at the end of the study and the other of which assumed that all such subjects were dead at the end of the study.

### Causes of death

A standardized death case report form was extracted from the SSTC database. Cause of death was then systematically verified against source documents. The causes of death were categorized as a single primary cause (either SSc or non-SSc-related) and all other SSc organ involvements that contributed to death. Death was attributed to SSc if the cause was identified with the specific organ involved. Death not attributed to SSc in the following cases:

- When the specific organ involved was cited in a diagnosis prior to SSc diagnosis, *except typical scleroderma involvement*
- Sudden inexplicable death
- Death from events with no direct link to SSc.

### Statistical analysis

Data are presented as the mean ± SD for continuous variables, the median and interquartile range for non-normally distributed continuous variables, and the number and percent for categorical variables. Baseline characteristics were compared using ANOVA variance analysis for continuous variables, and a chi-square test or Fisher’s exact test for categorical variables depending on the sample size.

Survival analysis was performed using the Kaplan-Meier method with comparisons performed using the log rank test. The primary end point was death from any cause or data censoring. The follow-up period ended in May 30, 2018.

Univariable and multivariable Cox proportional hazards models (ascending step-by-step method) were used to determine baseline variables associated with mortality. Variables with *p* value ≤0.05 in univariate analysis were selected for multivariate analysis. Analysis of the co-linearities variables were used to multivariable Cox model: PAPs >35 mmHg and Raynaud’s syndrome appearing after age 45 were not taken into account in the multi-variate analysis given the co-linearities respectively with the DLCO< 70% of the theoretical value and disease onset after the age of 50 years old.

Two-tailed *P* values less than or equal to 0.05 were considered significant. All statistical analyses were performed using SAS® software (French version 9.4).

## Results

### Characteristics of the population

**Fig. 1:**
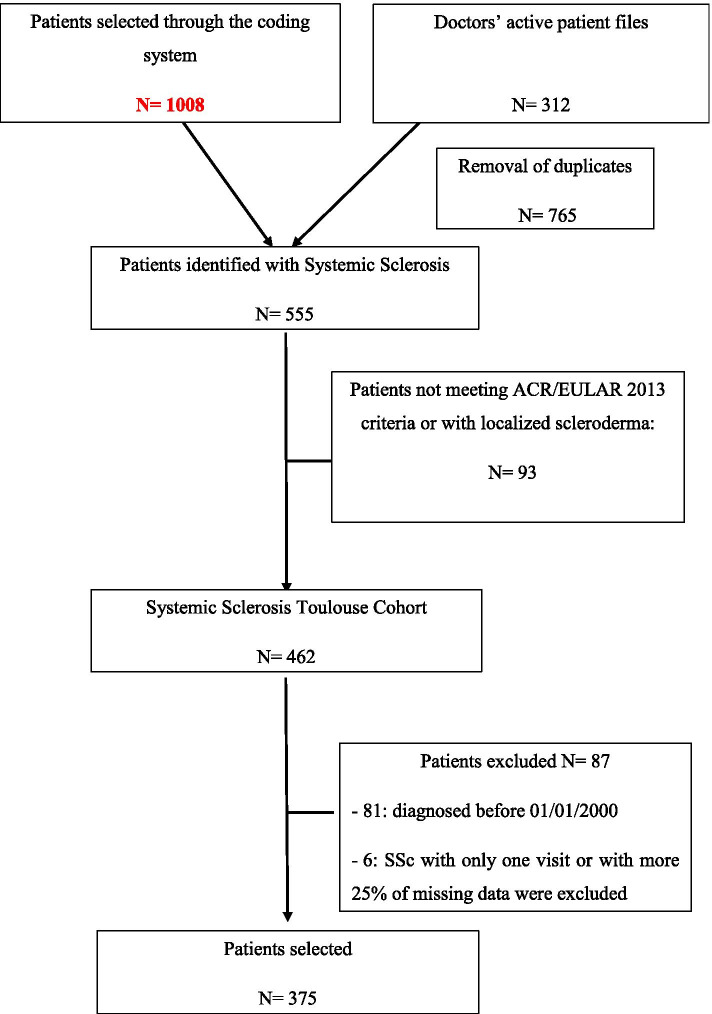
Flow chart showing selecting patients from Systemic Scleroderma Toulouse Cohort

We included 375 patients (292 females): 63 (15.2%) with diffuse cutaneous SSc, 279 (76.7%) with limited cutaneous SSc and 33 (8.1%) with SSc sine scleroderma between January 1, 2000, and January 1, 2018 (Fig. 1). The characteristics of patients at disease onset are shown in Table 1.

**Table 1:** Demographics and clinical characteristics of patients from systemic Scleroderma Toulouse cohort at baseline

	Complete cohort ***n***=375	Diffuse cutaneous SSc ***n***=63	Limited cutaneous SSc ***n***=279	Sine scleroderma ***n***=33
**Demographics**				
Female sex	292 (77.9)	38 (60)	226 (81)	28 (85)
Ethnic Group				
European	256 (89.8)	40 (80)	190 (91.3)	26 (96.3)
African	21 (7.3)	9 (18)	11 (5.3)	1 (3.7)
Asiatic	8 (2.8)	1 (2)	7 (3.4)	0
Smoking	129 (39.3)	23 (42.6)	95 (38.9)	11 (36.6)
BMI (kg/m^2^)	23.8 ±4.8	23.9 ±4.5	23.8 ±4.9	23.2 ±3.8
Age at disease onset	55.3 ±14.2	50.8 ± 13.4	56.4 ± 14	55.1 ± 15.4
Age at first Raynaud’s syndrome	45.9 ±16.6	48 ±13.6	45.9 ±16.9	43.5 ±17.8
Disease duration (years)	1.8 ±2.5	2.1 ±2.8	1.8 ±2.6	1.3 ±1.6
**Raynaud’s syndrome**	362 (96.5)	60 (95.2)	268 (96)	33 (100)
**Skin involvement**				
mRSS	6.8 ±9.4	19.2 ±11.1	5.3 ±6.3	0
Calcinosis	46 (12.4)	10 (15.9)	33 (12)	3 (9)
Telangiectasias	141 (38)	14 (22.2)	102 (37.1)	25 (75.7)
Active digital ulcers	57 (15.2)	7 (11.1)	48 (17.2)	3 (9)
Previously reported digital ulcers	53 (14.2)	11 (17.5)	38 (13.7)	4 (12.1)
**Cardiac involvement**				
ECG				
Bundle branch block	21 (6.1)	6 (10.5)	14 (5.4)	1 (3.2)
Arrhythmia	29 (8.4)	7/57 (12.3)	21/258 (8)	1 (3.3)
TTE				
LVEF (in %) +/- SD	66 ±9	65.6 ±9	66 ±9	67 ±7
Diastolic dysfunction	17 (5.6)	1 (1.8)	15 (6.7)	1 (4.2)
Pericarditis	18 (5.8)	7 (12.5)	9 (3.9)	2 (8.3)
Valvular disease	63 (20.4)	7 (12.5)	50 (21.8)	6/ (25)
**PH**				
PAPs ≥ 35 mm Hg (TTE)	64 (36.9)	12 (40)	47 (34.3)	5 (41.7)
PH (Right Catheterisation)	18 (4.8)	4 (6.4)	11 (4.2)	3 (9.3)
**Lung involvement**				
Interstitial syndrome	91 (25.4)	22 (34.5)	66 (25)	3 (9.3)
FVC < 70%	36 (12)	11 (20)	24 (10.9)	1 (3.8)
DLCO <70%	140 (46.9)	36 (65.4)	96 (43.8)	8 (33.3)
**Digestive tract involvement**	243 (64.8)	47 (74.6)	180 (64.5)	16 (48.5)
**Kidney involvement**				
Renal crisis	9 (2.4)	3 (4.7)	5 (1.8)	1 (3)
**Rheumatological involvement**				
Muscular signs	66	19 (30.1)	43 (15.4)	4 (12.1)
Joint signs	171 (45.6)	37 (58.7)	124 (44.4)	10 (30.3)
**Neurological involvement**	89 (23.7)	17 (26.9)	65 (23.2)	7 (21.2)
**Autoantibodies**				
Anti-centromere	185 (50.5)	7 (11.6)	152 (55.6)	26 (78.8)
Anti-Scl70	87 (23.8)	30 (48.4)	53 (19.6)	4 (12.1)
Anti-RNA pol III	16 (4.3)	4 (6.4)	12 (4.4)	0
Anti-U1RNP	4 (1.1)	2 (3.2)	2 (0.7)	0
Anti-PMScl	7 (1.9)	0	6 (2.2)	1 (3)
Anti-TIF1y	2 (0.5)	0	2 (0.7)	0
Anti-SSA	20 (5.5)	2 (3.2)	18 (6.6)	0
Anti-SSB	12 (3.2)	1 (1.6)	11 (4)	0
ANCA	5 (2.1)	1/41 (2.4)	4/169 (2.4)	0/21
**Laboratory parameters**				
Hemoglobin (g/dl),	13.2 ±1.5	13.1 ±1.6	13.2 ±1.5	13.5 ±1.4
Anemia <12 g/dl	54 (15.7)	15 (25)	35 (13.8)	4 (13.3)
CRP >5 mg/l	103 (32.8)	24 (43.6)	72 (31.2)	7 (25)
Albumin <35 g/l	34 (12.3)	10 (20.8)	23 (11.3)	1 (4.3)
**Drug exposure**				
Calcium channel blockers	213 (56.8)	39 (61.9)	156 (55.9)	18 (54.5)
Immunosuppressants				
Corticosteroids	135 (36)	41 (65.1)	92 (32.9)	2 (6.1)
Other immunosuppressant	120 (32)	38 (60.3)	78 (27.9)	4 (12.1)
Cyclophosphamide	44 (36.6)	18 (47.3)	25 (32)	1 (25)
Methotrexate	59 (49.1)	14 (36.8)	43 (55.1)	2 (50)
Azathioprine	25 (20.8)	7 (18.4)	17 (21.8)	1 (25)
Mycophenolate	62 (51.6)	27 (71)	34 (43.6)	1 (25)
Rituximab	20 (16.6)	4 (10.5)	13 (16.6)	0

Results are expressed as numbers (percentages) for qualitative variables and mean ±SD for quantitative variables. Arrythmia was defined as atrial or ventricular arrythmia

*ACAN* antinuclear antibodies, *ANCA* anti-neutrophil cytoplasmic antibodies, *Anti-RNA pol III* anti-polymerase III antibody, *BMI* body mass index, *DLCO* carbon monoxide diffusing capacity of the lung (% of predicted), *ECG* electrocardiogram, *FVC* forced vital capacity (% of predicted), *LVEF* left ventricular ejection fraction, *PAPs* systolic pulmonary arterial pressure, *PH* pulmonary hypertension, *SSc* systemic sclerosis, *mRSS* Rodnan modified skin score, *SD* standard deviation, *TTE* trans-thoracic echography

### SMR and survival analysis

During the study period, 69 patients died (18.4%) and 6 patients (only women, one sine scleroderma sub-type and 5 diffuse SSc) were lost to follow-up. The mean ± SD age at the time of death was 69.1 ± 14.8 years for SSc patients, 72 ± 15.5 and 64.2 ± 12.3 years, for women and men, respectively. The age—and sex-adjusted SMR of the cohort was 1.88 (95% CI 1.46–1.97) assuming that all subjects lost to follow-up were alive or 2.04 (95% CI 1.60–2.13) assuming that they were dead. Age-adjusted SMR for men was 3.61 (95% CI 2.35–3.94). Age-adjusted SMR for women was 1.80 (95% CI 1.31–1.92) assuming that all women lost to follow-up were alive or 2.05 (95% CI 1.52–2.18) assuming that they were dead. Age- and sex-adjusted SMR for diffuse subtype was 3.31 (95% CI 1.88–3.76). Age- and sex-adjusted SMR for limited cutaneous and sine scleroderma subtype was 1.74 (95% CI 1.30–1.85) and 1.03 (95% CI 0.20–1.49), respectively, assuming that all patients lost to follow-up were alive or 1.91 (95% CI 1.44–1.98) and 1.37 (95% CI 0.36–1.86) assuming that they were dead.

**Fig. 2:**
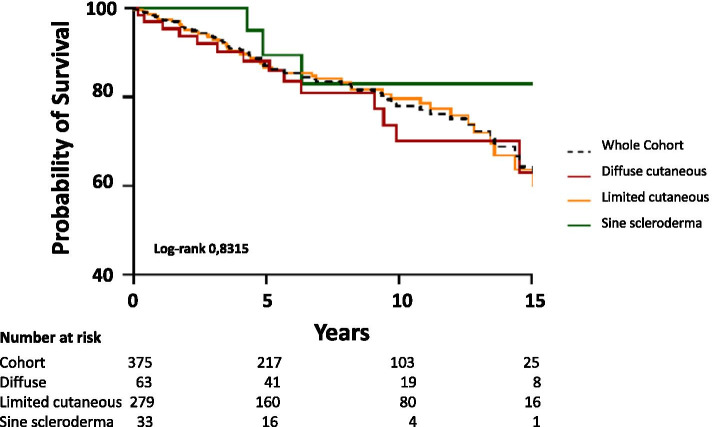
Kaplan-Meier analysis of overall survival following disease onset in the Systemic Scleroderma Toulouse Cohort according to SSc sub-types

The overall survival rates were 97.6% at 1 year, 93.4% at 3 years, 87.1% at 5 years, 77.9% at 10 years, and 61.3% at 15 years (Fig. 2). Survival for patients with SSc sine scleroderma tended to be the best. Indeed, the survival rates were for diffuse, limited cutaneous, and sine scleroderma sub-types 95.1%, 97.4%, 100% at 1 year; 89.8, 93, and 100% at 3 years; 85.5, 86.6, and 88.9% at 5 years; 69.7, 78.6, and 81.9% at 10 years; and 54.9, 59.7, and 81.9% at 15 years, respectively (Fig. 2).

### Predictors of mortality

Univariable Cox hazards analyses showed that subjects with male sex, Raynaud’s phenomenon onset after the age of 45, disease onset after the age of 50, cardiac involvement, PAPs > 35 mm Hg, DLCO <70%, FVC <65%, SRC, anemia, CRP > 5 mg/l, and albumin < 35 g/l had a higher risk of death (see Appendix). Multivariable Cox hazards regression analysis for all mortality in the cohort showed that male sex (HR=3.25 95%CI 1.69–6.22; *p*=0.0004), cardiac involvement (HR=2.86 95%CI 1.54–5.41; *p*=0.0012), DLCO<70% (HR=3.01 95%CI 1.40–6.88; *p*=0.0053), and a CRP >5 mg/l (HR=2.13 95%CI 1.11–5.41; *p*=0.0174) were independent predictors of risk (Table 2). The results of the two sensitivity analyses performed to account for predictors of mortality at 5 and 10 years, respectively, were consistent with those of the primary analysis (Table 2).

**Table 2:** Multivariable predictors of mortality in the systemic Scleroderma Toulouse cohort at 5, 10 years, and all mortality

	Multivariate 5 years			Multivariate 10 years			All mortality		
	HR	95% CI	*p*	HR	95% CI	*p*	HR	*95% CI*	*p*
**Sex: male**	2.131	0.99–4.58	0.0526	3.42	1.75–6.67	0.0003	3.25	1.69–6.22	0.0004
**DLCO <70%**	5.489	1.61–18.60	0.0063	4.54	1.74–1.85	0.002	3.1	1.40–6.88	0.0053
**Cardiac involvement**	2.893	1.30–6.41	0.0089	3.18	1.62–6.24	0.0008	2.86	1.52–5.41	0.0012
**CRP >5 mg/l**	3.283	1.42–7.53	0.005	2.37	1.23–6.460	0.01	2.13	1.14–5.41	0.0174

The results are expressed as hazard ratios (HR) with a 95% confidence interval (95% CI), cardiac involvement comprises left ventricle ejection fraction <50%, and/or a TTE anomaly (pericarditis, valvular disease, or diastolic dysfunction), and/or an ECG anomaly (arrhythmia or conduction blocks). PAPs >35 mm Hg and Raynaud’s syndrome appearing after age 45 were not taken into account in the multi-variate analysis given the co-linearities respectively with the DLCO< 70% of the theoretical value and disease onset after the age of 50 years old

### Causes of death

**Fig. 3:**
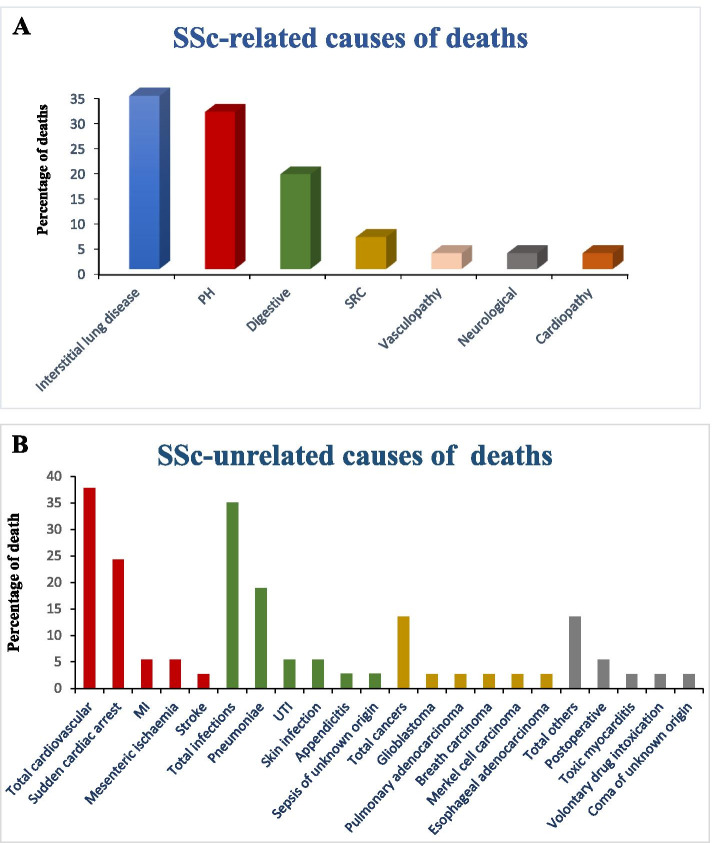
Primary causes of deaths in Systemic Scleroderma Toulouse Cohort. **A** Percentages of causes of death related to systemic sclerosis. **B** Percentages of causes of death non-related to systemic sclerosis. The bars in bold correspond to the groups of causes of death and in the same color are represented the different etiologies. MI myocardial infarction, PH pulmonary hypertension, SSc systemic sclerosis, UTI urinary tract infection

Between January 2000 and May 2018, there were 69 deaths (42 [62.3%] women). Death was considered SSc-related in 32 cases (46.4%) and unrelated to SSc in 37 cases (53.6%). Among the SSc-related death, three main causes were identified: ILD (34.4%), PH (31.2%), and GI involvement (18.8%) (Fig. 3A). The most common causes of non-SSc-related death were cardiovascular events (37.8%) and infections (35.1%) well in front of malignancies (13.5%) (Fig. 3B). Thus, the principal causes of non-SSc-related death were cardiovascular with sudden cardiac arrest (24.3%), myocardial infarction (5.4%), and mesenteric ischemia (5.4%). Pneumonia predominated among the infections.

## Discussion

This study still clearly confirmed an increased mortality risk from SSc, compared with the general healthy population. This is one of the only to describe the mortality rate of SSc sine scleroderma. Male sex, cardiac involvement, systemic inflammation, and altered DLCO were independent mortality risk factors. The majority of deaths (53.6%) was not attributed to SSc directly, especially cardiovascular mortality (37.8%).

Our SMR is lower than those produced in the two most recent meta-analyses focusing on mortality in SSc. Rubio-Rivas et al. [8], analyzing 17 articles from 1964 to 2005, found an SMR of 2.72 (95% CI 1.93–3.83). More recently, Pokeerbux et al. [11] reported an SMR of 5.73 (95% CI 4.68–6.94) from a French multi-centered cohort of patients suffering from SSc. The latter also carried out a meta-analysis of 18 articles with a pooled SMR of 3.45 (95% CI 3.03–3.94). However, the literature reveals a wide variability of mortality rate in SSc, with SMRs ranging from 1.34 to 7.18 [8, 10, 11, 17]. Such variability is probably caused by considerable methodological differences in the various studies carried out, differences in time origin from which survival time is calculated and this disease high heterogeneity. The elevated SMR figure in the Pokeerbux et al. cohort study [11] could also be explain by the over-representation of patients with anti-SCL70 antibodies (35% in their study vs. 23.8% in our cohort). Other explanations could account for our SMR within the lower range of published data such as a lower number of diffuse cutaneous subtype of SSc (16.8% vs. 23.4 to 46%) [8–11, 19, 40–44], a lower number of patients with a SRC (2.4% compared to 2.9–10%) [9–11, 19, 40, 44], and a less extended level of cutaneous involvement (average Rodnan skin score of 6.8 for the patients in the present study vs. 9) [11]. Regarding SRC, we described the same incidence as in the recent German epidemiological study evaluating renal involvement on 2873 patients with SSc [50]. In addition, we kept in our analysis SSc sine scleroderma, which is often excluded from such studies that could explain our relatively low SMR. Indeed, sine scleroderma subtype tended to have the best survival in our study with more 80% of survival rate at 15 years; the same result was found by Siméon-Aznar et al. [51]*.* The SMR of our cohort more approximated “mortality with real life,” taking into account the different subtypes of SSc, in particular sine scleroderma subtype.

Our overall survival rates at 1 year, 3 years, 5 years, and 10 years were consistent with those reported previously [8, 9, 11, 23, 26]. In addition, we have shown a non-significant higher mortality rate from the diffuse cutaneous sub-type vs. the limited cutaneous subtype and the sine scleroderma subtype of SSc, in line with the published literature [8, 11, 18, 24, 25, 52–54]. However, the absence of significance may be related to an underpowered sample size for this analysis.

Our study identified 4 independent predictors of mortality: male sex, cardiac involvement, DLCO <70% of predicted, and CRP >5 mg/ml, which were consistent with previous studies [11, 23, 52, 55–61]. Indeed, a low DLCO in SSc often associated with ILD and/or PH (essentially pulmonary arterial hypertension and secondary pulmonary hypertension resulting from ILD), the two main causes of SSc-related mortality. Male SSc patients often presented with a worst prognostic than female SSc patients [11, 60, 62, 63], like in our cohort. Cardiac involvement during the course of SSc is also associated with an increased risk of mortality, especially pericardial effusion [11, 57, 59, 64], valvular disease [11], and an LVEF <50% [23]. In our study, cardiac involvement was a composite outcome including LVEF <50%, and/or a TTE anomaly (pericarditis, valvulopathy, or diastolic dysfunction), and/or an ECG anomaly (arrhythmia or conduction blocks), which was strongly associated with a 2.9-fold increase mortality risk (HR=2.86 95% CI 1.54–5.41). Finally, we confirmed that systemic inflammation was a major pejorative prognostic factor of mortality (HR 2.13 95%CI 1.11–5.41) like reported by few studies [11, 23, 53]. Diffuse subtype does not appear as a prognosis factor; this result can be explained by an underpowered sample size of diffuse and anti-SCL70 subtypes for this analysis. In several studies, diffuse subtype is a strong prognosis factor. Thus, in EUSTAR cohort, diffuse subtype does not appear to be a major factor in mortality (HR 0.79 (0.67 to 0.93)) [12]. In the SCleroderma mOrtality p Eustar (SCOpE), a score predicting mortality from EUstar cohort, diffuse subtype was one of the least weighted criteria after DLCO<60%, age, dyspnea, LVEF <50%, FVC<70%, and renal involment [12]. As already discussed, the incidence of this complication has decreased, and in our cohort, we have a lower rate of this involvement that others studies. It was a possible explanation that diffuse subtype is not a risk factor for mortality in our study.

One of the most interesting results of our study was that the majority of identified death in the SSTC cohort was not related to SSc directly. Due to a better understanding of SSc and a better management of this severe systemic disease, SSc-related deaths became less frequent [3, 18]. The first cause of non-SSc related in our work was cardiovascular diseases in a higher proportion than in previous large cohort studies (2–11.9%) [8, 9, 18, 62]. The cardiovascular-related deaths are represented mainly by sudden cardiac arrest. The majority of cardiac arrest cases, occurring at the patient’s home, were diagnosed by general practitioner and thus could be subject to a bias. However, 46.4% of the deaths in our cohort were directly attributable to SSc. Our results were consistent with the data from the EUSTAR cohort, finding that nowadays, the two main causes of SSc-related were ILD and PH [3, 6, 18, 23, 25, 52, 53, 62]. SSc GI-related mortality was frequent in our cohort (18.2%), and this cause of death represented 7.6% of deaths in metanalysis of Rubi-Rivas et al. [8]. Due to our strict methodology with a thorough medical chart review and systematic telephone tracing of patient’s general practitioner to ascertain cause of death, we were perhaps more able to identify this complication and its prognosis.

The major strengths of the present study are that we carried out an exhaustive medical chart review for each included patient, which led to detailed clinical and laboratory characteristics in a large cohort of incident patients. We ascertained vital status and cause of death by systematically interrogated the SSTC database, reviewed each medical chart a second time, called each patient’s GP, and interrogated the Registre d’état civil [47]. Other major strengths of this study are the long-term follow-up of patients, a very low attrition rate, as only six patients were lost to follow-up covering a period of 18 years and that we kept for the analysis SSc sine scleroderma which is often excluded from clinical trial and cohort studies. Thus, this study provides the sine scleroderma subtype survival. Probably, our overall SMR is more pertinent, not excluding the subtypes.

Οur study has some limitations. Our study is necessarily limited by its retrospective and bi-centric nature. Because it is a retrospective study, misclassification of systemic sclerosis subtypes is possible and induces a classification bias. Additionally, we did not include information about the treatments used during the follow-up due to heterogeneity in time of treatment initiation and the duration or type of treatment administered and as a consequence the effect of the various treatments in the natural course of the disease could not be eradicated. Concerning causes of deaths, potential interactions between SSc-related and unrelated to SSc may exist; this distinction is sometimes not perfectly clear. However, our results are coherent with results in others studies, like EUSTAR cohort. Finally, as with all the large cohorts collected over many years, there is the bias of survival. The longer the period covered, the greater is the risk of losing track of dead patients, and thus survival data could be distorted.

## Conclusion

In conclusion, our results show that mortality is still substantial in SSc despite constant therapeutic progress. This is one the first study that ascertained SSc sine scleroderma mortality rate alongside the two other sub type of SSc. This serves as an important reference for future survival analysis and epidemiology surveys. Our study identifies strong predictors of mortality male sex, cardiac involvement, DLCO <70%, and CRP > 5 mg/l. Non-SSc-related death is more frequent than SSc-related deaths, of whom cardiovascular disease is the most common. An early and systematic management of the large proportion of cardiac complications is in order, in hope of extending SSc outcome.

## Supplementary Information

**Additional file 1: Appendix.** Prognosis factors: COX univariate analysis in the Systemic Scleroderma Toulouse Cohort

## Data Availability

Data are available in a public, open access repository.

## Abbreviations

ACA: Anti-centromere
ANA: Antinuclear
ATS: American Thoracic Society
BMI: Body mass index
CNIL: Commission Nationale Informatique et Liberté
DLCO: Diffusing capacity of the lung for carbon monoxide (% predicted value)
ECG: Electrocardiogram
ERS: European Respiratory Society
FVC: Forced vital capacity (% predicted value)
GI: Gastrointestinal
HRCT: High resolution computed tomography
ILD: Interstitial lung disease
INSEE: Institut national de la statistique et des études économiques
LVEF: Left ventricular ejection fraction
mRSS: Modified Rodnan skin score
PAH: Pulmonary arterial hypertension
PASP: Pulmonary arterial systolic pressure
PAWP: Pulmonary artery wedge pressure
PH: Pulmonary hypertension
RP: Raynaud’s phenomena
SD: Standard deviation
SMR: Standardized mortality ratio
SRC: Scleroderma renal crisis
SSc: Systemic sclerosis
SSTC: Systemic Scleroderma Toulouse Cohort
TRV: Tricuspid regurgitation velocity
TTE: Trans-thoracic echogragraphy
