# Supplementary material for: Sine scleroderma, limited cutaneous, and diffused cutaneous systemic sclerosis survival and predictors of mortality
Source: Arthritis Res Ther. 2021 Dec 7;23:295. doi: 10.1186/s13075-021-02672-y (PMC8650544; doi:10.1186/s13075-021-02672-y)
Supplement: Supplementary file 1 — Additional file 1: Appendix. Prognosis factors: COX univariate analysis in the Systemic Scleroderma Toulouse Cohort [file 13075_2021_2672_MOESM1_ESM.pdf]

Appendix: Prognosis factors: COX univariate analysis in the Systemic Scleroderma Toulouse Cohort

	Univariate analyses		
	HR	95% CI	p
**Form of systemic sclerosis**			
Diffuse SSc vs. limited cutaneous forms	1.21	0.68-2.15	0.5167
**Demographic data**			
Sex: male	3.10	1.88- 5.12	<0.0001
African vs. European	1.12	0.40-3.11	0.8235
Smoking	1.19	0.72-1.95	0.4883
BMI <18	1.57	0.67-3.66	0.29
Raynaud's syndrome after 45 years	2.96	1.49-5.86	0.0018
Disease onset disease after 50 years	3.06	1.64-5.72	0.0004
**Skin involvement**			
mRSS > 15	1.59	0.78-3.28	0.2016
Puffy fingers	0.61	0.32-1.16	0.1326
Sclerodactyly	1.43	0.87-2.35	0.1605
Calcinosis	0.92	0.44-1.93	0.8344
Telangiectasias	1.41	0.87-2.25	0.1607
Ulcers (history of ulcers, active ulcers, pulpy scars)	0.88	0.50-1.54	0.6454
**Cardiac involvement**			
Cardiac involvement (TTE anomalies and/or ECG)	3.37	1.99-5.69	<0.001
**PH**			
PAPs ≥ 35 mm Hg (TTE)	2.77	1.44-5.32	0.002
**Kidney involvement**			
Scleroderma renal crisis	6.75	2.41-18.94	0.0003
**Lung involvement**			
FVC < 70%	1.63	0.81-3.24	0.1652
DLCO <70%	5.06	2.58-9.52	<0.0001

**Neurological involvement**	1.1	0.64-1.89	0.7238
**Blood tests**			
Anti-centromere	1.11	0.68-1.81	0.6764
Anti-Scl70	1.25	0.74-2.13	0.3937
Anti-RNA polymerase III	0.79	0.10-5.81	0.8225
Anti-PMScl	0.87	0.12-6.26	0.8858
Anaemia <12 g/dl	2.92	1.68-5.1	0.0001
CRP >5 mg/l	4.03	2.37-6.83	<0.0001
Albumin <35 mg/dl	1.24	1.03-1.51	0.024

The results are expressed as Hazard ratios (HR) with a 95% confidence interval (95% CI) Cardiac involvement comprises left ventricle ejection fraction <50%, and/or a TTE anomaly (pericarditis, valvular disease or diastolic dysfunction), and/or an ECG anomaly (arrhythmia or conduction blocks). BMI: Body mass index; DLCO: pulmonary diffusing capacity (% of predicted); FVC: forced vital capacity (% of predicted); PAPs: systolic pulmonary arterial pressure; PFT: pulmonary function tests; SSc: systemic sclerosis; mRSS: Rodnan modified skin score TTE: transthoracic echography.
